# A novel blood-feeding detoxification pathway in *Nippostrongylus brasiliensis* L3 reveals a potential checkpoint for arresting hookworm development

**DOI:** 10.1371/journal.ppat.1006931

**Published:** 2018-03-22

**Authors:** Tiffany Bouchery, Kara Filbey, Amy Shepherd, Jodie Chandler, Deepa Patel, Alfonso Schmidt, Mali Camberis, Adeline Peignier, Adam A. T. Smith, Karen Johnston, Gavin Painter, Mark Pearson, Paul Giacomin, Alex Loukas, Maria-Elena Bottazzi, Peter Hotez, Graham LeGros

**Affiliations:** 1 Malaghan Institute of Medical Research, Wellington, New Zealand; 2 The Ferrier Research Institute, Victoria University of Wellington, Lower Hutt, Wellington, New Zealand; 3 Centre for Biodiscovery and Molecular Development of Therapeutics, Australian Institute for Tropical Health and Medicine, James Cook University, Cairns, Queensland, Australia; 4 Departments of Pediatrics and Molecular Virology and Microbiology, National School of Tropical Medicine, Baylor College of Medicine, Houston, Texas, United States of America; 5 Texas Children's Hospital Center for Vaccine Development, Houston, Texas, United States of America; 6 Department of Biology, Baylor University, Waco, Texas, United States of America; Imperial College London, UNITED KINGDOM

## Abstract

As part of on-going efforts to control hookworm infection, the “human hookworm vaccine initiative” has recognised blood feeding as a feasible therapeutic target for inducing immunity against hookworm infection. To this end, molecular approaches have been used to identify candidate targets, such as *Necator americanus (Na)* haemoglobinase aspartic protease-1 (APR-1), with immunogenicity profiled in canine and hamster models. We sought to accelerate the immune analysis of these identified therapeutic targets by developing an appropriate mouse model. Here we demonstrate that *Nippostrongylus brasiliensis (Nb)*, a phylogenetically distant strongylid nematode of rodents, begins blood feeding early in its development and that immunisation with *Na*-APR-1 can block its growth and completion of its life cycle. Furthermore, we identify a new haem detoxification pathway in *Nb* required for blood feeding that can be blocked by drugs of the quinolone family, reducing both infection burden and the associated anaemia in rodents. Collectively, our findings show that haem metabolism has potential as a checkpoint for interrupting hookworm development in early stages of the hookworm life cycle and that the *Nippostrongylus brasiliensis* rodent model is relevant for identifying novel therapeutic targets against human hookworm.

## Author summary

Hookworm infections (*Necator americanus* or *Ancylostoma duodenale*) represent a major neglected tropical disease affecting approximately 450 million people worldwide and causing morbidity due to their need to feed on host blood resulting in severe anemia. New chemotherapy and vaccines are needed to combat hookworm infections. Using a rodent parasite model, we describe a new haem detoxification pathway that is a metabolic checkpoint for parasite development, survival and reproduction. This provides a starting point for the development of novel therapies against such metazoan blood-feeders.

## Introduction

Hookworms (Ancylostomatoidea) are agents of one of the major Neglected Tropical Diseases, affecting 450 million people worldwide [1]. Human hookworm disease is caused principally by *Na* and *A*. *duodenale* and manifests as anaemia through blood-loss, stunted development in childhood and complications during pregnancy [2, 3]. Blood-loss is thought to be associated with the feeding activity of the parasite in the gut throughout the L4 and adult stages, during which the parasite attaches to the gut mucosa and ruptures capillaries. The blood-feeding mechanisms have been partially characterised in these nematodes, and some proteins involved in this pathway such as the *Na* hemoglobinase aspartic protease 1 (*Na*-APR-1) and the haem transporter *Na* gluthatione-S-transferase-1 (*Na*-GST-1), that are essential to the digestion process, are now the targets of vaccine development [4–7].

Haem, an essential prosthetic group, is one of the byproducts of the degradation of haemoglobin. Most nematode parasites lack the *de novo* production of haem and are as such dependent on haem scavenging from the host [8]. However, haem in its free form is highly toxic, and its detoxification is essential to the survival of haematophagous parasites [8]. This process has been partially studied in hookworms with the discovery of a haem catabolism pathway involving the GST and GSH proteins, similar to that described for the malaria parasites *Plasmodium* spp. and other haematophagous parasites [9–12]. In malaria, several pathways of haem detoxification have been described. One of these pathways involves the crystallisation of haem into a β-haematin complex called hemozoin [13, 14]. Hemozoin is a dark-brown non-toxic pigment and has been characterised in both *Plasmodium* spp. and in the blood flukes *Schistosoma* spp. [15]. Given the presence of hemozoin in such distantly related parasites, we hypothesized its possible formation in hookworms.

As human hookworms do not develop in mice, we used a phylogenetically distant strongylid nematode that is widely used to study the type 2 immune response, namely *Nb* (Trichostrongyloidea). This parasite has a similar life cycle to *Na*, migrating from the skin to the lungs during the infective L3 stage (iL3), and maturing to adulthood in the gut from where it releases eggs into the faeces. Larvae can be found in the lungs approximately 11 hours post-subcutaneous infection. There, they enter the 3^rd^ molt that differentiates them from the L3 to the L4 stage in around 48 hours. This ecdysis is rarely observed in the lungs, but all the larvae that reach the gut by 72 hours are L4. The morphological changes associated with the 3^rd^ molt are considerable, and can be summarized as follows: significant growth of the larvae (increasing more in width than in length), shortening and widening of the buccal cavity, increase in length of the oesophagus, increase in number and widening of intestinal cells, and accumulation of a dark-brown intestinal pigment [16].

We designed *in vitro* and *in vivo* assays to demonstrate that *Nb* is a haematophagous parasite from the iL3 stage to the adult stage, causing anaemia in its host just as described in human hookworm infection. We have shown that the uptake of RBC, or of haemoglobin, induces growth of the parasite and the formation of a dark brown pigment that we characterized as hemozoin-like. Drugs of the quinolone family targeting hemozoin formation are able to arrest the development of the iL3 and the reproductive capacity of the adults both *in vitro* and *in vivo*.

## Results

### *N*. *brasiliensis* is a blood-feeding nematode from its infective larval stage

**Fig 1:**
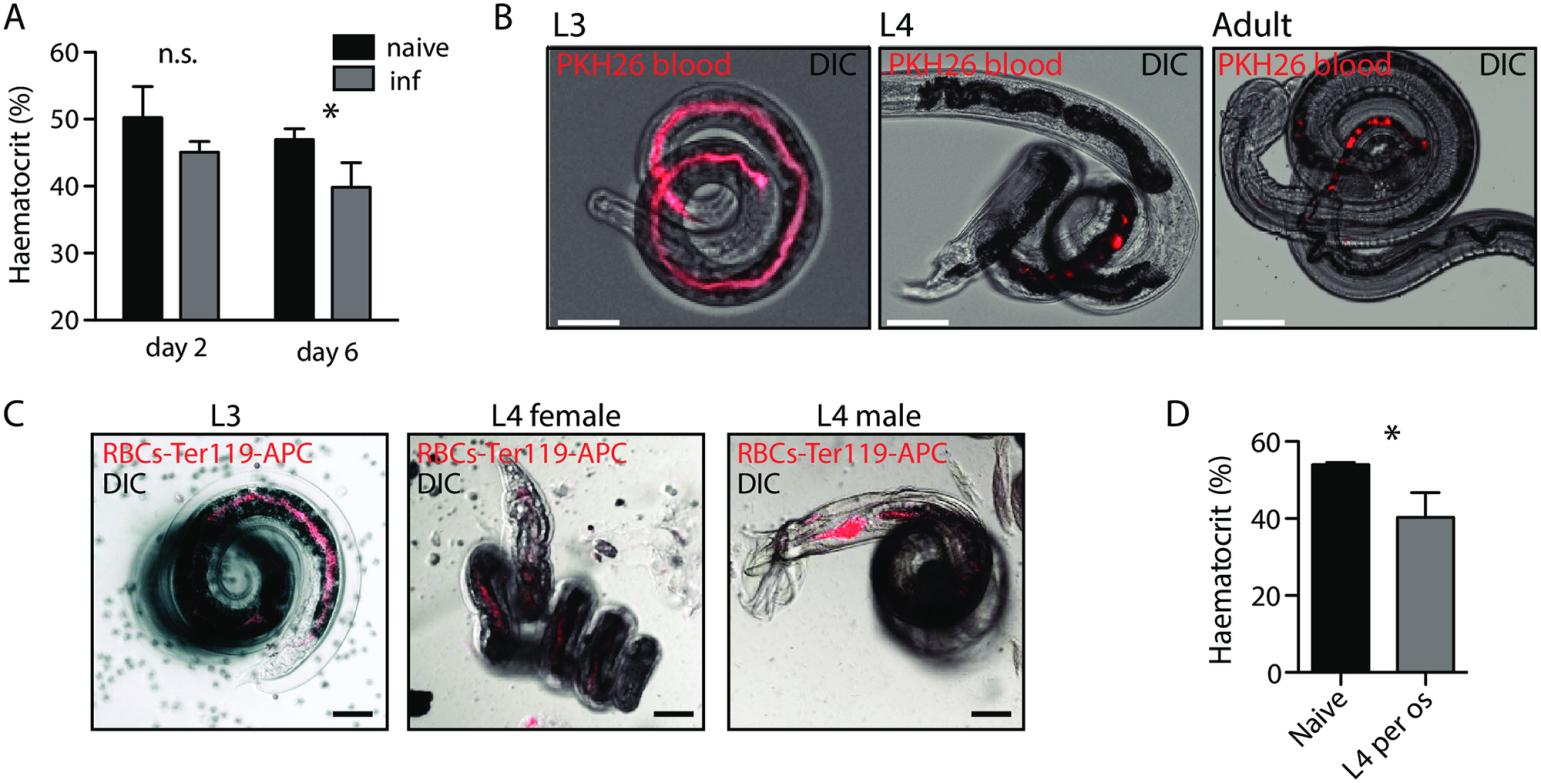
*Nb* is a blood-feeding nematode and causes anaemia. (A) Haematocrit was measured at day 2 and day 6 on 30 μl blood from C57BL/6 mice infected subcutaneously with *Nb*. One-way ANOVA (n = 6, pool of 2 independent experiments). (B) PKH26 was administered intravenously to C57BL/6 mice, one day before collection of L3 (1 day post-infection), L4 (3 days post-infection) or adult *Nb* (6 days post-infection). One day later, parasites were harvested and gut fluorescence assessed by fluorescent and DIC imaging. Images representative of 50 larvae harvested in 3 independent experiments. Scale bar: 50 μm. (C) 15 μg of Ter119-APC antibody was administered intravenously to C57BL/6 mice, daily, starting one day before infection with 550 iL3 *Nb*. Larvae were harvested from the lung 1 day after infection, and female and male L4 from the gut at day 4. Fluorescence of the larval intestine was assessed by fluorescent and DIC imaging. Representative of 30 larvae harvested in 2 independent experiments. Scale bar: 50 μm (D) Haematocrit was measured at day 6 on 30 μl blood from C57BL/6 mice infected with L4 *Nb per os*. t-test.

Anaemia is the main pathology associated with hookworm infection and an important cause of adverse pregnancy outcomes and developmental stunting in children in endemic areas [2, 3]. Here, we report that mice infected with *Nb* develop a mild anaemia, during both the lung phase and the gut phase of the parasite life cycle (Fig 1A). As anaemia could be due to a combination of lung damage caused by the worm migration [17] and damage through feeding in the gut, we sought direct evidence of parasite ingestion of blood by using fluorescently-labelled whole blood or Ter-119-labelled red blood cells (RBC). In all stages tested (iL3 in lungs, L4 or adults in the gut) we observed fluorescence in the intestine of the parasite, confirming that *Nb* is ingesting blood *in vivo* (Fig 1B & 1C). We further confirmed that infection by gavage of L4, which is not a migratory stage, also causes anaemia (Fig 1D).

### The blood digestome of *N*. *brasiliensis* is conserved with human hookworms

**Fig 2:**
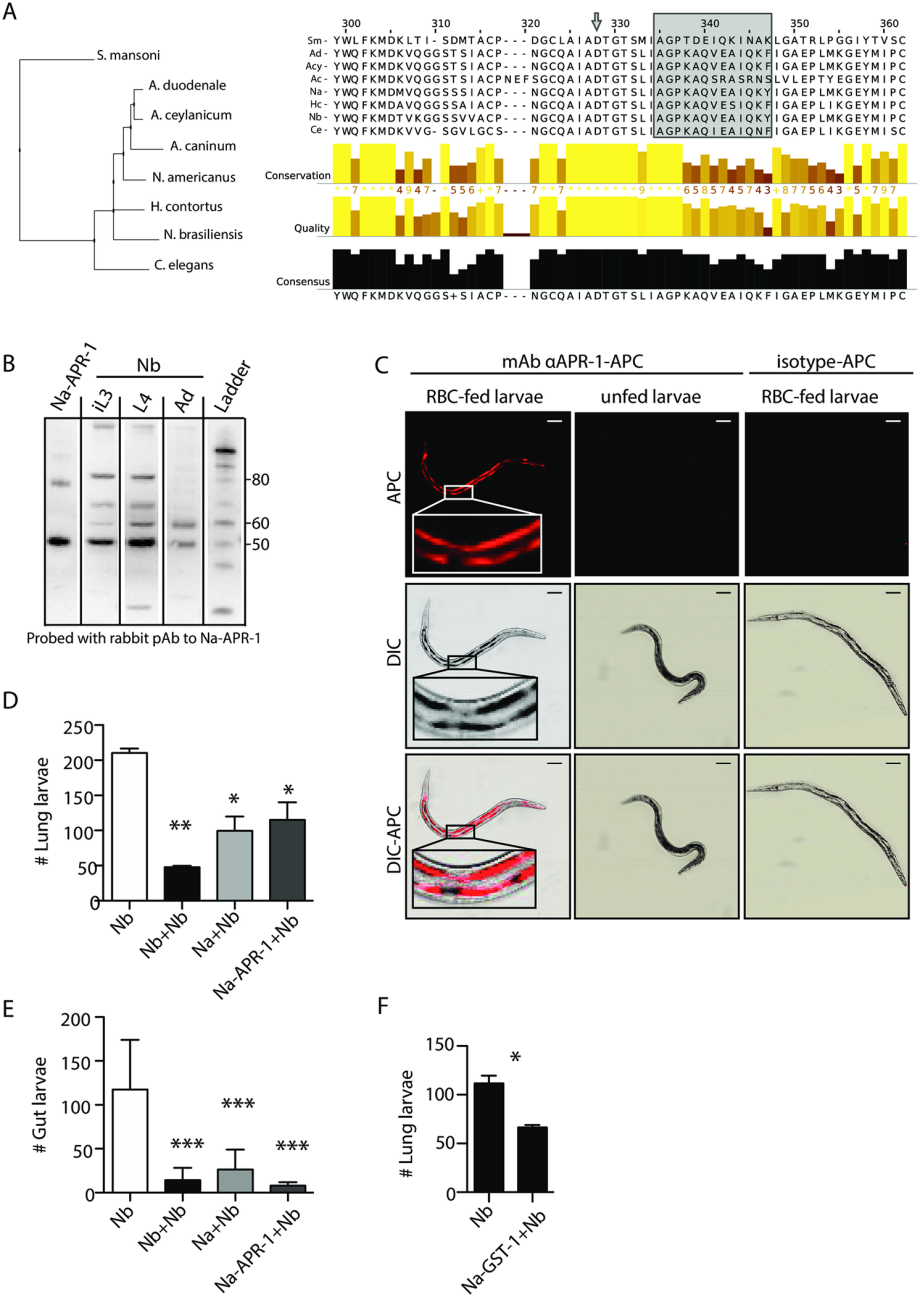
The *Nb* haemoglobin digestion cascade is conserved with human hookworms. (A) Amino acid alignment of *Nb*-APR-1 and the corresponding regions of other parasite and aspartic protease homologues. The active-site Asp284 is denoted by an arrow and the 13-mer A291Y epitope of *Na*-APR-1, and its corresponding peptide in homologous proteases is shown in the grey box. GenBank accession numbers are as follows: *N*. *americanus*, CAC00543; *A*. *ceylanicum*, AAO22152; *A*. *duodenale*, ACI04532; *Ancylostoma caninum*, AAB06575; *Schistosoma mansoni*, AAB63442; *Haemonchus contortus*, CDJ87729 and *Caenorhabditis elegans*, NP510191. *Nb* sequence (m.418883) is available in S1 Text. (B) Western blot of *Nb*-APR-1 expression in crude extracts of *Nb* iL3, L4 or adults. r*Na*-APR-1 is included as a reference. Representative of 4 pools of *Nb*. (C) iL3 were fed *in vitro* for 24 hr with RBC and treated with 10 μg of 11F3-APC monoclonal antibody against *Na*-APR-1 or with an isotype matched-RELM-APC control antibody. Larvae were allowed to empty their digestive contents for 2 hours in fresh DMEM before internal labelling was evaluated by confocal microscopy. Data representative of 50 larvae cultured in 3 independent experiments. Scale bar: 50 μm (D&E) Mice were vaccinated subcutaneously with 25 μg of *Na*-APR-1 combined with Alu-Gel-S (25 μg: 200 μl), or infected with 100 *Nb* or *Na* iL3 by intravenous administration. One month later, mice were challenged with 550 *Nb* iL3 subcutaneously and larvae were enumerated in the lung 2 days post-inoculation (D) or 6 days later in the gut (E). Data representative of two independent experiments (n = 3–5), one–way ANOVA. (F) Mice were vaccinated with 25 μg of *Na*-GST-1 intraperitoneally or infected with 100 *Nb* or *Na* iL3 by intravenous administration. One month later, mice were challenged with 550 *Nb* iL3 subcutaneously and larvae were enumerated in the lung 2 days post-inoculation. Data representative of two independent experiments (n = 5), t-test.

In order to confirm the blood-feeding behaviour of *Nb*, we searched for homologues of *Na*-APR-1, the first enzyme of the haemoglobin digestion cascade in *Na* [18] within the *Nb* secretome and transcriptome [19]. Amongst the excretory-secretory products of *Nb* iL3 and adult stages, we identified a potential homologue of *Na*-APR-1, hereafter *Nb*-APR-1, presenting 83% amino-acid identity over 91% of the protein length, notably including a conserved active site (Fig 2A, S1 Text). A protein-based neighbour-joining phylogram of several homologues from related organisms confirms the proximity of the *Nb*-APR-1 homologue to those of the hookworm family (Fig 2A).

We next explored the pattern of expression of *Nb*-APR-1 throughout the *Nb* life cycle by western blot using a monoclonal antibody raised against the *Na*-APR-1 protein, as its binding site was fully conserved between both species [5](Fig 2B). We found that *Nb*-APR-1 is expressed in both gut stages (L4 and adults) but also, surprisingly, in the iL3. In *Necator*, such expression of APR-1 in the iL3 stage has not been reported, although APR-1 mRNA has been detected [20].

Next, we assessed whether anti-APR-1 antibody could bind to APR-1 in live parasites, the expression of which is restricted to the nematode intestine [7]. By culturing serum-activated *Nb* iL3 *in vitro* with an anti-APR-1 antibody in the presence or absence of murine blood, we show that the antibody is naturally ingested by the parasite and binds to the intestinal brush border (Fig 2C). Remarkably, we did not observe any binding of the antibody to the intestinal border in unfed iL3, suggesting that *Nb*-APR-1 could be transported to the luminal surface upon initiation of blood feeding (Fig 2C). As APR-1 is shared between the rodent hookworm *Nb* and the human hookworm *Na*, we assessed whether there was an immune cross-reactivity between these pathogens. Even though *Na* is unable to develop into L4 in an incompatible host such as the mouse [21], intravenous injection of *Na* iL3 was sufficient to induce protection against *Nb* both during the lung and the gut stages (Fig 2D & 2E). More strikingly, vaccination with a wild-type *Na*-APR-1 recombinant protein formulation with alum was sufficient to elicit protection against *Nb*, even as early as the lung phase of the infection (Fig 2D & 2E).

To further validate the relevance of *Nb* as a vaccine target discovery model for hookworms, we assessed the cross-protection potential of *Na*-GST-1, the other lead vaccine candidate against hookworms, thought to be involved in haem detoxification [22–25]. First, we identified 11 homologous sequences with a high identity (>55%) to *Na*-GST-1 in the *Nb* transcriptome (S1 Fig)[19]. Two of those proteins are secreted by the adult stage but not the iL3 (m.154242 and m.83139), similar to the pattern of expression described in *A*. *caninum* [9, 26]. Importantly, intraperitoneal vaccination with recombinant *Na*-GST-1 with alum also conferred protection against the lung stage of *Nb* infection (Fig 2F).

Altogether, the blood-feeding-induced anaemia and conservation of the molecular blood-feeding pathways in *Nb* show that this parasite can be used as a relevant tool for vaccine and drug identification against hookworms.

### *N*. *brasiliensis* infectious larvae ingest blood and develop a haem-derived pigment

In hookworms, blood-feeding was thought to be restricted to the adult stage, with its main role being to support the reproduction of the parasites [3]. However, APR-1 is expressed in the iL3 stage, raising the possibility that hookworms are blood-feeders throughout their parasitic life cycle, and as such could be targeted by vaccination earlier than previously suggested [27]. This prospect is supported by mRNA analysis of *Na* [20]. We thus investigated the potential importance of blood-feeding in the development of iL3.

**Fig 3:**
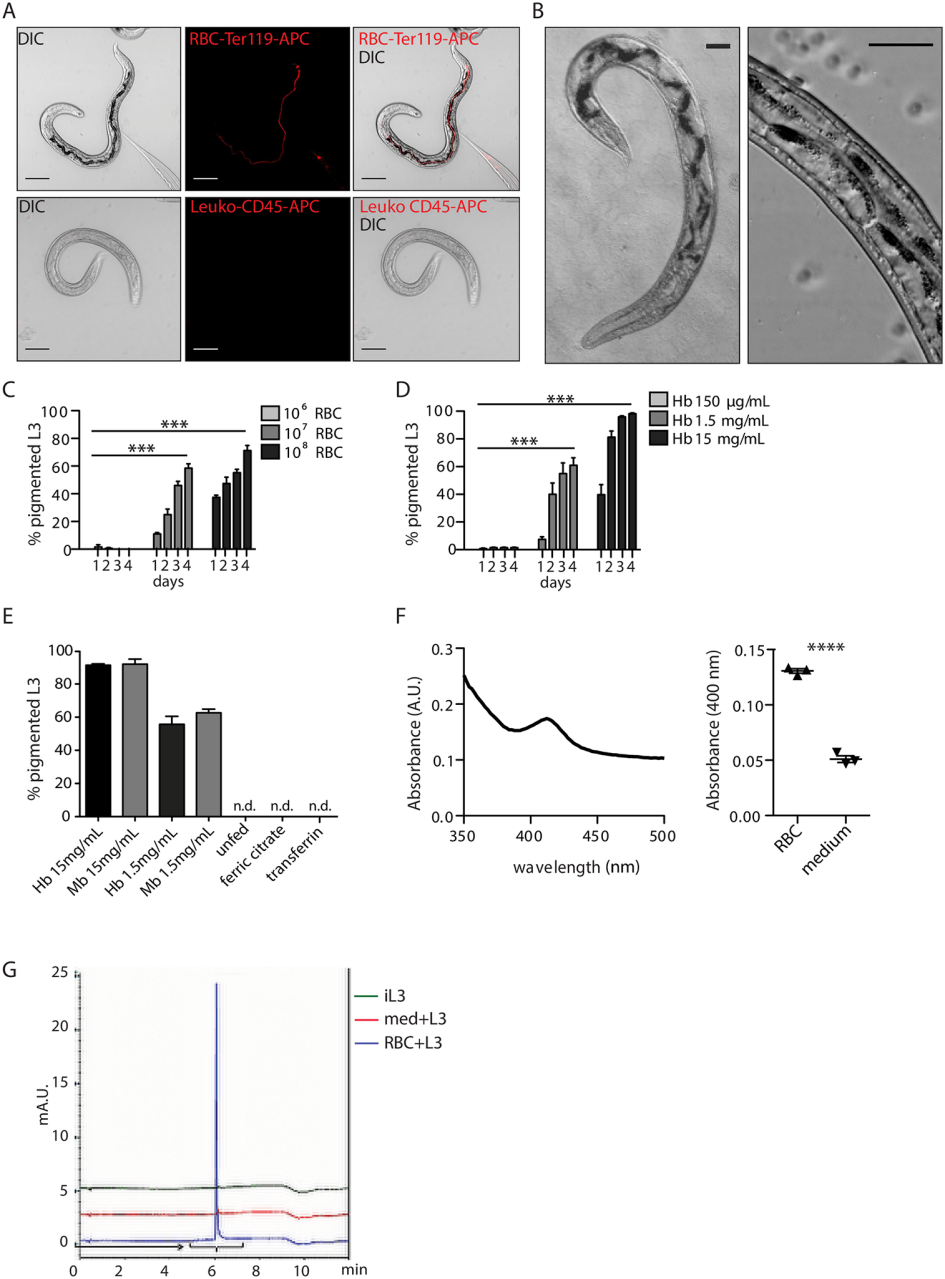
*Nb* detoxify blood in an hemozoin-like pigment contained in intestinal cells. (A) RBC or spleen leukocytes were isolated and stained with Ter119-APC or CD45-APC antibody respectively. Cells were then co-cultured at 1x10^8^ cells per 1500 iL3 for 24 hours after which larvae were assessed for internal fluorescence by confocal imaging. Data representative of two independent experiments, with at least 50 larvae observed for each experiment. Scale bar: 50 μm. (B) DIC image of iL3 *in vitro* fed with RBC for 48 hours, showing the intracellular localisation of the pigment in the epithelial cells of the parasite intestine. Data representative of three experiments, at least 50 larvae observed for each experiment. Scale bar: 25 μm. (C) 1500 iL3 are cultured *in vitro* in the presence of increasing doses of purified RBC. The percentage of larvae harbouring intestinal pigmentation was observed on days 1–4. Data representative of three independent experiments, two-way ANOVA significant for both time and dose effect, Bonferonni post-test significant for all time points relative to 10^6^ RBC. (D) 1500 iL3 are cultured *in vitro* in the presence of increasing doses of human haemoglobin (Hb), at concentrations equivalent to the dose of haemoglobin per RBC reported in (C). The percentage of larvae harbouring intestinal pigmentation was observed on days 1–4. Data representative of three independent experiments, two-way ANOVA significant for both time and dose effect, Bonferonni post-test significant for all time points relative to 150 μg/mL of haemoglobin. (E) 1500 iL3 are cultured *in vitro* for 48 hours in the presence of human haemoglobin (Hb), human myoglobin (Mb), transferrin (3.68 mg/mL) or ferric citrate (22.5 μg/mL), all at the same relative proportion of iron. The percentage of larvae harbouring intestinal pigmentation was counted at 48 hours after stimulation. *n*.*d*. = not detected. Data representative of three independent experiments. See S2B Fig. (F) The specific absorbance spectrum of larvae dissolved in 0.1 M NaOH after 48 hours of feeding with 10^8^ RBC. Differential absorbance at 400 nm between RBC-fed iL3 and unfed iL3. Data representative of three independent experiments, t-test. (G) LC-MS spectrum showing specific peak for haem at 616.17 g/mol in the pigment fraction extracted from *in vitro* RBC fed larvae for 48 hours (RBC + L3). Control show inactivated larvae (iL3) and larvae culture in medium only (med. +L3).

First, we show that *Nb* iL3 can ingest blood and that this feeding is specific to RBC, as only Ter119-labelled RBC and not CD45-labelled leukocytes are ingested by the parasite (Fig 3A). Using intravital imaging of the parasite we additionally observed the movement of a RBC bolus in the intestine of the worms (S1 Video). Within 24 hours of RBC-feeding *in vitro*, *Nb* iL3 develop a dark brown pigmentation that clearly accumulates inside the gut epithelial cells (Fig 3B). This is reminiscent of the characteristic intestinal pigmentation that appears in the lung molt 3 larvae both in *Nb* and *Na* [16, 28](S2A Fig). The percentage of iL3 presenting with this pigmentation through time *in vitro* increased in a dose-dependent manner with RBC number, and with haemoglobin concentration (Fig 3C & 3D). Interestingly, myoglobin but not other iron-carrier proteins (hemin, hematin nor transferrin) caused development of pigmentation in the parasite, suggesting that the blood digestion cascade is very specific and similar to that of hookworms (Fig 3E, S2B Fig).

As the intestinal pigment appears specifically after RBC or haemoglobin feeding of *Nb* iL3, we hypothesised that the pigment could be part of a haem detoxification pathway. Microscopic examination of RBC-fed iL3 identified the black pigment within the digestive tract as birefringent, a feature consistent with crystals of haem [29]. The haem nature of the pigment was further confirmed by its specific absorbance at 400 nm, and its presence only in RBC-fed larvae (Fig 3F). Liquid chromatography mass spectrometry (LC-MS) analysis of the purified pigment fraction (RBC+L3) identified a peak at 616.17 consistent with a haem B signature. LC-MS analysis of the isolated pigment fraction from unfed larvae (iL3) and medium-fed larvae (med. + iL3) did not present with this characteristic peak (Fig 3G). Taken together, these results suggest that the intestinal pigment forming in the *Nb* intestine after blood-feeding is a detoxified form of the haem released from haemoglobin digestion, similar to hemozoin described in other blood-feeding parasites [30, 31].

### Blood feeding is an important cue for development of *N*. *brasiliensis*

Hookworms cause extensive haemorrhage by damaging the lungs during their migration, and are thus exposed to potentially harmful haemoglobin that requires detoxification. We sought to establish if accumulation of the haem-derived pigment in iL3 *Nb* is an artefact of the haemorrhage caused by tissue damage or whether active blood-feeding is required for the development of iL3 into more mature stages.

**Fig 4:**
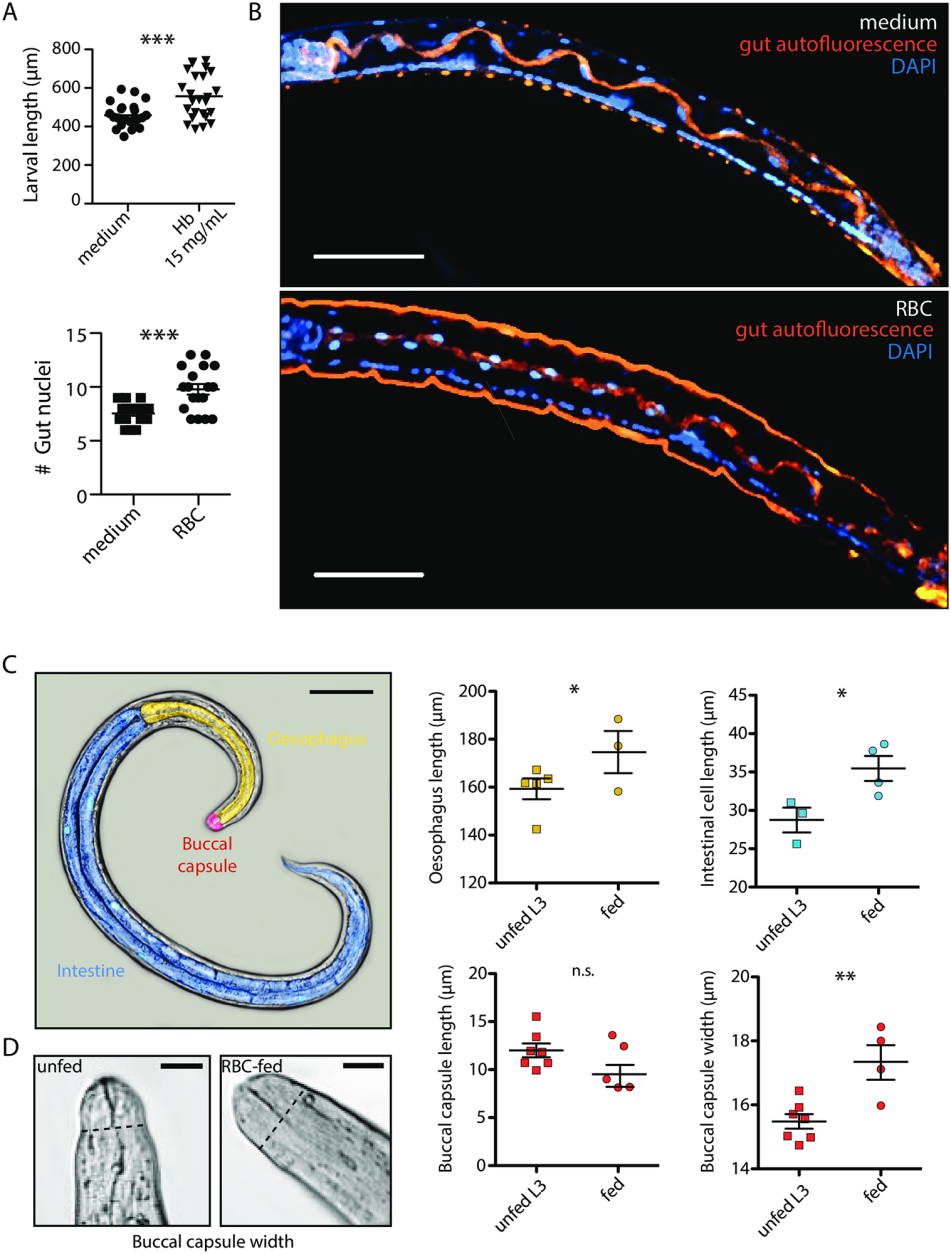
Blood feeding is required for optimal development of *Nb* L3. (A) Larval length (μm) was measured 4 days after the beginning of *in vitro* culture with 15 mg/mL of haemoglobin. Data pooled from three independent experiments, t-test. (B) Enumeration of nuclei in the intestine of *Nb* iL3 fed for 48 hours with RBCs *in vitro* or unfed (medium) iL3. After 48 hours of feeding, the larvae were fixed in 2% formalin for 72 hours and stained with DAPI overnight with gentle agitation. DAPI nuclear staining (blue) and the autofluorescence from the cuticle and from the intestinal lumen (orange) were assessed by confocal imaging, by focusing on the central plane of the transparent larvae to observe the intestine. Nuclei from one side of the intestine were counted. Data pooled from 2 independent experiments (n = 18), t-test. Scale bar: 50 μm. (C) Pseudo colouring of an unfed exsheathed iL3 cultured *in vitro* for 4 days. The buccal capsule is represented in red, the oesophagus in yellow and the intestine in blue. The dimensions of these three distinct morphological compartments were measured in unfed or RBC fed larvae. Data representative of two independent experiments (n = 3–7 worms), t-test. Scale bar: 100 μm. (D) DIC images of the buccal capsule of *in vitro* RBC-fed or unfed larvae after 4 days of culture. Dotted line represents where the buccal capsule width was measured. Scale bar: 10 μm.

First, we show that *in vitro* feeding of iL3 with haemoglobin is sufficient for the larvae to grow in size (Fig 4A). Using fluorescent staining of nuclei in whole mount *Nb* iL3, we further show an increase in differentiation of the intestine with a proliferation of the intestinal epithelial cells, from 8 to 12 cells (Fig 4B). We further addressed whether other signs of molt 3 in the parasite were initiated after blood consumption. In accordance with the molt 3 specific morphological criteria described for *Nb* [16], we observed (i) an increase of the overall size of the larvae (Fig 4A), (ii) the elongation of the oesophagus, (iii) the increase in the length of the intestinal cells, and finally (iv) the widening and shortening of the buccal capsule after RBC or haemoglobin feeding (Fig 4C & 4D).

In summary, blood-feeding is essential for the early development of *Nb* larvae. We thus considered whether haem metabolism could be a checkpoint in worm development that could be leveraged to control infection.

### The ingested haem is detoxified into a hemozoin-like pigment that can be targeted by quinoline drugs

The pigment we identified in *Nb* presents very similar physiochemical characteristics to hemozoin, a haem-detoxification crystal that has been described in other blood-feeding organisms, such as the protozoan *Plasmodium* and the trematode *S*. *mansoni* [30, 31].

**Fig 5:**
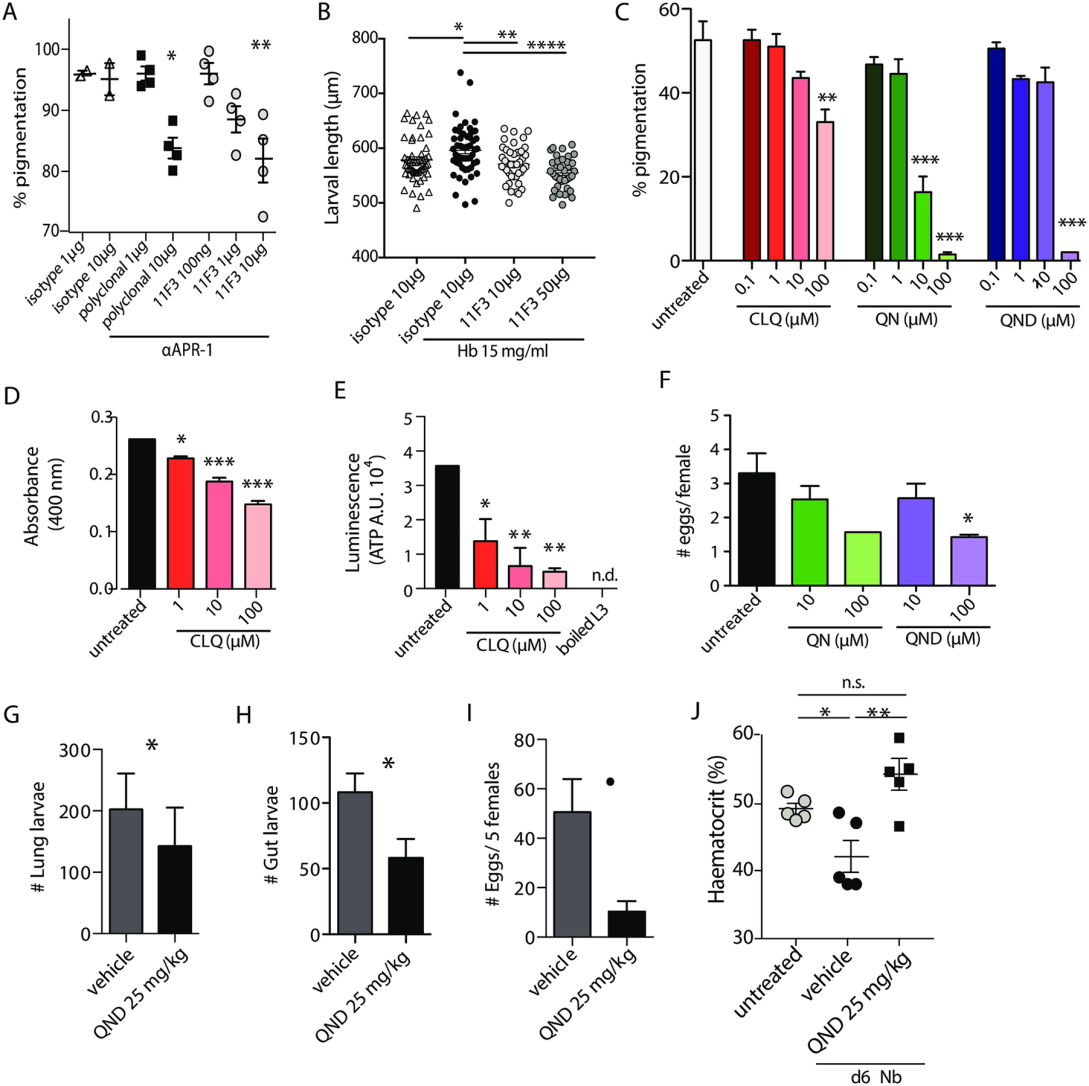
Hemozoin detoxification in hookworms can be targeted by quinolones. (A) 1500 iL3 were fed *in vitro* with RBC and treated with increasing doses of 11F3 monoclonal antibody against *Na*-APR-1, a polyclonal antibody against *Na*-APR-1, an isotype-matched control antibody or left untreated. The percentage of larvae with internal pigmentation was evaluated 4 days later. Data representative of three independent experiments, one-way ANOVA. (B) 1500 iL3 were fed *in vitro* with 15 mg/mL haemoglobin and treated with increasing doses of 11F3 monoclonal antibody against *Na*-APR-1, an isotype-matched control antibody or left untreated. The length of larvae was measured after 5 days in culture. Data representative of two independent experiments with 50 larvae per treatment measured, t-test. (C) iL3 were fed *in vitro* with RBC with increasing doses of quinine (QN), chloroquine (CLQ) and quinidine (QND), and the percentage of larvae with an internal pigmentation was evaluated 4 days later. Data representative of three independent experiments (with triplicates of 1500 iL3 for each experiment), one-way ANOVA, Bonferonni post-test compared to untreated. (D) Absorbance at 400 nm of iL3 fed *in vitro* with RBC for 4 days with or without CLQ. Data representative of three independent experiments (with triplicates of 1500 iL3 for each experiment), one-way ANOVA, Bonferonni post-test compared to untreated (E) ATP measurement of iL3 fed *in vitro* with RBC for 4 days with or without CLQ. Boiled iL3 were used as a negative control. Data representative of two independent experiments (with triplicates of 100 iL3 for each experiment), one-way ANOVA, Bonferonni post-test compared to untreated. (F) Adults were isolated from rat intestines 10–12 days post subcutaneous infection with *Nb*. 100 males and 100 females were co-cultured with 10^8^ RBC in the presence of QN or QND for a week, in triplicate wells. The number of eggs released during that time was counted by salt flotation. Data representative of three pooled experiments, two-way ANOVA, Bonferonni post-test compared to untreated. See S3D Fig. (G)&(H) Worm burden in the lung at 2 days (G) and the gut at 6 days (H) post-infection after daily intraperitoneal injections with 25 mg/kg QND, or vehicle alone. Data representative of three independent experiments, t-test (n = 5–6) (I) Fecundity of female *Nb* treated *in vivo* for 6 days with QND, assessed by number of eggs released *in vitro* for 48 hours post recovery from the intestine. Data representative of two independent experiments, t-test (n = 5–6). (J) Haematocrit was measured at day 6 on 30 μl blood from C57BL/6 mice infected subcutaneously with *Nb* and treated daily with 25 mg/kg QND intraperitoneally. Data representative of two independent experiments, t-test (n = 5–6).

Hemozoin formation in *S*. *mansoni* is prevented by RNAi blockade of cathepsin D, the homologue of APR-1 in this trematode [32]. Consistent with the possibility of a hemozoin-like pigment in *Nb*, APR-1 blockade with a monoclonal antibody prevents pigment formation in *Nb* iL3 (Fig 5A). Furthermore, iL3 fed with RBC *in vitro* and fasted for 48 hours lose their pigmentation, suggesting that hemozoin could also be a form of iron storage as described in *S*. *japonicum* [33](S3A Fig). Additionally, anti-APR-1 (mAb 11F3) blocks larval growth after feeding with haemoglobin, in a dose-dependent manner (Fig 5B). Together, these results suggest that *Nb* possesses an alternate haem detoxification pathway to GST that could lead to new drug/vaccine targeting in hookworm infection.

To further evaluate whether the hemozoin-like pigment in *Nb* iL3 is the product of a haem detoxification pathway, we carried out pigment formation inhibition experiments using quinolines, compounds that have been described as specifically able to target the formation of hemozoin in both malaria and Schistosoma [34, 35] [36]. Consistent with our hypothesis, chloroquine (CLQ), quinine (QN) and quinidine (QND) were all able to block pigment formation in *Nb* iL3 in a dose-dependent manner (Fig 5C). Associated with the reduction in the proportion of worms that develop the intestinal pigmentation, we observed a diminution of pigment intensity per worm (Fig 5D) and a decrease in worm viability as measured by ATP levels (Fig 5E). Notably, none of the drugs caused toxicity to the parasite in the absence of blood-feeding (S3B Fig).

As the intestinal pigment was also identified in adult stages (S3C Fig), we next assessed the effect of blocking the haem detoxification in sexually mature adults. In line with our observations with iL3, we noticed a diminution in worm pigmentation and fecundity after quinine treatment in a dose-dependent manner (Fig 5F, S3D Fig). To confirm that the hemozoin-like pigment could be a useful target against hookworms, we assessed the effect of blocking the haem detoxification pathway in hookworms *in vivo* in mice. We administered QND intraperitoneally at 25 mg/kg daily, and assessed *Nb* worm burden 2 days post-infection in the lungs and 6 days post-infection in the gut, thus investigating both the developing larvae and the sexually mature parasite. As expected, QND treatment caused a significant reduction in worm burden at both 2 and 6 days post-infection, as well as a significant reduction in numbers of eggs released by the adults (Fig 5G–5I). More strikingly, the mild anaemia caused by *Nb* compared to uninfected control mice, was also countered by QND treatment (Fig 5J). Altogether, these results show haem metabolism to be a promising target for both vaccine-based and chemotherapeutic hookworm interventions.

## Discussion

Hookworms are considered to cause one of the major neglected tropical diseases, and infect around 450 million people worldwide [1]. The human disease is caused principally by either *N*. *americanus* or *A*. *duodenale* infection, and is characterized clinically by anaemia, malnutrition in pregnant women, and an impairment of cognitive development in children [2, 37, 38]. While the host immune response to hookworm infection is robust and comprehensive in scope, activating both strong humoral and cellular responses, it fails to elicit protection against subsequent infection, highlighting the need for design of an efficient vaccine [27, 39].

In this work we re-describe a well-known murine model of hookworm infection in light of new insights into its life cycle. *Nb*, which belongs to the Strongylida order and yet is phylogenetically distant from human hookworms, has so far been used only as a surrogate for understanding the immune response against hookworms, given that the parasite was believed to be non-blood-feeding [40]. Here we demonstrate that, contrary to those beliefs, *Nb* is indeed a blood-feeding nematode and that its haemoglobin digestion cascade is conserved with *Na* and *A*. *duodenale*.

Notably, we report that *Nb* is blood-feeding early in its lifecycle with the APR-1 protein being expressed in the iL3 stage. Due to the difficulty of accessing human hookworm material the proteomes of *Na* and *Ancylostoma* spp. are still presently unavailable, but *APR-1* RNA expression has been identified in the iL3 stage of both *Na* and the zoonotic hookworm *A*. *caninum* [20]. While it is currently accepted that hookworms are blood-feeding only from their intestinal stage (L4 onwards), other proteins involved in the blood-feeding cascade, such as the saposin-like protein of *A*. *caninum* that allows the lysis of RBC in the parasite intestine, have been reported to be expressed in the iL3 stage [41]. Furthermore, it has been described that *Na* and *Nb* iL3 cultivated *in vitro* in chicken embryo extract develop a similar intestinal pigmentation to the one we report here after feeding *Nb* iL3 with haemoglobin [28, 42]. Altogether this raises the possibility that hookworms may, in fact, be blood-feeding as early as the infective larval stage. Interestingly, the amino acid sequence for *Nb*-APR-1 shows homology to sequences for aspartic proteases found in other helminth species, including those known to be non-blood-feeding, such as the free-living nematode *C*. *elegans* and the gut-dwelling rodent helminth *Heligmosomoides polygyrus*, suggesting that these molecules have a general role in protein turnover regardless of the food source. Further experiments to assess the effects, if any, of anti-APR-1 on *H*. *polygyrus* larval development *in vivo* could elucidate its role in this helminth which is thought to graze on epithelial cells, and not use RBC as its primary food source [43].

The rationale for the design of an efficient vaccine against helminths includes combining antigens from both the infectious stage (to limit establishment of the parasite) and the adult stage (to alleviate the pathology and create a reproductive bottleneck). Previous attempts at vaccination with ASP-2, the lead antigen targeting hookworm iL3 stages, did not live up to expectations when clinically tested in humans [44, 45], thus highlighting the need for novel targets against this stage. We believe that vaccination with APR-1 or other proteins of the blood digestome could ensure the blockade of hookworm development at not only the reproductive stage, but also the establishment stage. Indeed, targeting blood-feeding is now the major strategy of the human hookworm vaccine initiative, with two blood-feeding antigens (*Na*-APR-1 and *Na*-GST-1) now in human clinical trials [46–48]. Our study highlights the importance of further dissection of the molecular pathways in the blood-feeding cascade, using high-throughput approaches such as RNA-seq and proteomics, to discover new blood-feeding-targeting drugs or vaccines of therapeutic potential.

One important extrapolation from our data is that quinolones, well-known for their anti-malarial activity, could also target the hemozoin-like pigment arising from the blood-feeding behaviour of human hookworm parasites. While chemotherapy is currently the treatment of choice to control helminth infection, of the four available treatments against soil-transmitted helminths, only albendazole produces satisfying protection [49]. More worryingly, such drugs have well-known limitations: single dose regimens are inefficient, re-infection occurs rapidly after treatment, and drug resistance can arise (as shown in veterinary medicine) [50–52]. Consequently, research to develop and maintain a pipeline of new anthelmintic drugs in addition to specific anti-hookworm vaccines to prevent/limit infection is indispensable. While quinolones cannot be candidates for direct use against helminths (due to widespread multi-resistances developed by *Plasmodium* spp.)[53, 54], their efficiency against the hookworm rodent model, *N*. *brasiliensis*, pinpoints a vulnerability in the parasite’s metabolism. Interestingly, a cross-epidemiological study of the effect of chloroquine treatment for malaria in hookworm-endemic areas previously established that treated patients presented a reduced egg burden and pathology [55], confirming *in situ* the relevance of targeting the haem detoxification pathway in hookworm infection. Furthermore, co-infections with hookworms and schistosomes are common, and there are at least a dozen countries (within both Africa, South America and Asia) with more than five million infection cases by each [56]. Due to both the important overlap of endemic regions of these parasites and the synergistic effect of their blood-feeding on anemia, a multivalent vaccine targeting both parasites would be a solution of choice [56]. The remarkable convergence of the blood digestome and haem detoxification via a hemozoin-like pigment in distant species such as *P*. *falciparum*, *S*. *japonicum* and *Nb* in particular, points to new avenues of research for the identification of multivalent vaccine antigens. It also raises the possibility that a drug targeting the hemozoin-like formation could be used to treat three of the most widespread and debilitating human infections at once.

In conclusion, our study describes the requirement for blood-feeding for the early development of gastrointestinal nematode larvae, which opens an opportunity to target the establishment of haematophagous helminths in the host through vaccination against the blood-feeding digestome, or chemotherapy such as drug administration of quinolones. This could potentially reduce the global impact of human blood-feeding nematodes such as hookworms and *Schistosoma* spp. Notably, such discoveries could also be transferred to veterinary medicine, helping to alleviate the economic and ecological burden of species such as *Haemonchus contortus* [57].

## Methods

### Mice

C57BL/6J mice used in these experiments were bred by the Biomedical Research Unit, Malaghan Institute of Medical Research, Wellington, New Zealand. Mice were used at 6-10-weeks old, and age- and sex-matched.

### Maintenance, isolation, use of iL3, L4 & adult stages of *N*. *brasiliensis*

*Nb* was originally sourced from Lindsay Dent (University of Adelaide) and has been maintained by monthly passage through Lewis rats for 20 years. iL3 larvae were prepared from 2-week rat fecal cultures and viable larvae were recovered from lung or gut tissue, all as previously described [58]. Briefly, tissue was diced, placed on cheese-cloth, and suspended in a 50 mL centrifuge tube containing PBS at 37°C for at least 2 h. Viable worms migrated out and accumulated at the bottom of the tube before being counted on a gridded counting plate. Day 0 always refers to the day of infection with *Nb*. Subcutaneous infections were performed by inoculating mice in the scruff of the neck with 550–600 live iL3 worms in a volume of 200 μl. L4 were harvested at day 3 in the gut; adults at day 6. For cross-protection experiments 100 iL3 of *Na* or *Nb* were administered intravenously 30 days before the subcutaneous challenge with 600 iL3 of *Nb*. Larvae were killed for some experiments by boiling them in the microwave for 5 minutes.

### *In vivo* treatments

For *in vivo* whole-blood labelling, mice were intravenously injected with 10 μl PKH26 cell membrane dye (Sigma) in 100 μl of diluent C (as per manufacturer’s recommendation), two days before parasite harvest. For *in vivo* labelling of RBC, 10 μg of anti-Ter-119-APC antibody (eBioscience) in PBS was intravenously administered to mice two days before parasite harvest.

For QND treatment, mice were injected intraperitoneally daily with either 25 mg/kg QND (Sigma) in 200 μl PBS containing 4% Tween 80 (Sigma) or vehicle alone, starting the day before infection with *Nb*. QND was made up fresh every two days. Haematocrit measurements were obtained by collecting blood directly into heparinised glass haematocrit capillary tubes (Vitrex Medical, Denmark) and centrifuging for 15 min at 1500 g. Packed cell volume was measured with a ruler to the nearest millimetre. Haematocrit was calculated as the percentage of packed red blood cells to the total length occupied by the packed red blood cells, the white blood cells and the plasma together.

For vaccination with wild-type r*Na*-APR-1 protein [4] or r*Na*-GST-1 protein [22] (both produced in a yeast expression platform, respectively lot A141112CJK-1 and lot G090513EMH-01, from the Sabin Vaccine Institute, TX), 25 μg of protein combined with alum (Alu-Gel-S, Serva, Germany) was administered subcutaneously (APR-1) or intraperitoneally (GST-1), each week for 3 weeks. Mice were allowed to rest for 15 days, before subcutaneous challenge with 600 iL3 of *Nb*.

### Protein purification and western blot

Whole worms were crushed in PBS and the homogenates centrifuged at 12,000 g at 4 °C for 30 minutes. The supernatants were taken, and protein concentration was quantified using a bicinchoninic acid assay. 20 μg of worm lysate and 0.1 μg of recombinant *Na*-APR1 protein were run on a 4–12% Bis-Tris gel. After transfer, membranes were probed with a polyclonal rabbit antibody generated against the recombinant *Na*-APR1 protein [5] overnight at 4 °C. For visualization, membranes were probed with a goat anti-rabbit HRP secondary antibody and visualized under luminescence with a Gel Logic 4000 Pro.

### Larvae *in vitro* culture

iL3 were washed several times in PBS and incubated for 1 hr at 37°C in an antibiotic solution (Penicillin/Streptomycin 10X (Gibco), Gentamicin 3X (Sigma) in PBS). 1500 larvae were then cultured overnight in complete DMEM (DMEM (Gibco) plus 10% FBS (Gibco), 1% L-glutamine (Gibco), 1% Penicillin/Streptomycin, 1% Gentamicin) in 12 or 24 well plates. Supplements (haemoglobin, myoglobin, transferrin, ferric citrate or haemocyanin (all Sigma)) were prepared in complete DMEM and added to the culture medium at concentrations according to the experiment. Quinolines were made fresh for every experiment (CLQ, QN, QND (all Sigma)) at 100 mM in complete DMEM.

RBC were collected by cardiac puncture or cheek vein bleeding the day of culture into Alsevers solution. They were then washed 3 times in RBC washing buffer [59], which resulted in 98% purity (as identified by Ter119 staining by flow cytometry). RBC were co-cultured in complete DMEM with 1500 iL3 at 1x10^8^/mL unless otherwise specified.

For *in vitro* blocking of larval pigmentation with the antibody to *Na*-APR-1, *Nb* iL3 were co-cultured in complete DMEM with or without RBCs for 48 hours in the presence of increasing doses of monoclonal antibody to *Na*-APR-1 (clone 11F3), an isotype matched control antibody or polyclonal antibodies to *Na*-APR-1 [5]. For blocking of growth, larvae were cultured in cDMEM alone or with 15 mg/mL haemoglobin (Sigma), with increasing doses of 11F3 for 5 days. Larvae were then washed, individual larvae imaged and measured to the nearest 0.001 μm using ImageJ. 50 larvae per treatment were measured.

Adult *Nb* were obtained from the guts of rats 10–12 days after infection, washed several times with PBS, then incubated for at least 1 hour at 37 °C in an antibiotic solution (Penicillin/Streptomycin 10X (Gibco), Gentamicin 3X, Neomycin 3X (Sigma) in PBS). 100 males and 100 females per well were cultured in 6 well plates for 7 days in DMEM, 10% FBS, 1% Neomycin, 1% Penicillin/Streptomycin and 1% Gentamicin with or without quinolines. Egg output was then quantified following the salt flotation technique using a McMaster egg slide chamber.

### Microscopy

The monoclonal anti-*Na*-APR-1 antibody (11F3) was coupled to APC, using APC-Lightning Link (Innovabiosciences) according to the manufacturer’s instructions. An isotype-matched antibody was similarly coupled to APC. Prior to imaging, worms were transferred into complete DMEM for 1 hour to allow intestinal contents to be expelled. The fluorescence was evaluated by confocal microscopy.

For RBC staining, aliquots of 1 million cells were stained with Ter119-APC or PKH26 (Sigma) according to manufacturer recommendations. White blood cells were obtained by passing mouse spleens through a 70 μm cell filter (BD Falcon). After RBC lysis, the cells were extensively washed in PBS with 1% FBS to limit haemoglobin contamination. Aliquots of 1 million cells were then stained with CD45-APC (BD Pharmingen) and co-cultured with 1500 iL3 at 1x10^8^/mL for 24 hours. Fluorescence in the gut of the larvae was evaluated by confocal microscopy 24 hours after the initiation of feeding.

Fluorescence was recorded with an FV1200 confocal microscope (Olympus, Japan). Samples were imaged through a 10x or 20x objective. For detection of fluorophores, samples were exposed to diode laser light at a wavelength of 559 nm for the excitation of PKH26, and 633 nm for the excitation of APC. The fluorescence was detected through 540/100 and 680/100 filters respectively. DAPI was excited by a 405 nm laser and was detected through a 445/15 filter. The larval gut autofluorescence can be detected after 635 nm excitation through a 705/50 filter. The intracellular localisation of the pigment was observed by differential interference contrast (DIC) imaging on a confocal using 633 nm excitation. Images were analyzed and color channels were merged with ImageJ [60].

### Larval morphology and viability

The following parasite features were analysed by DIC microscopy (larvae were fixed *in toto* with 2% formaldehyde (Sigma) in cold PBS to avoid body shrinkage): i) size of the larvae, ii) size of the oesophagus, iii) the length and width of the buccal capsule, iv) the length of the intestinal cells. For pigment quantification larvae were observed live, as fixation destroys the pigment. For larval viability measurements, 20 larvae were homogenized using 1.1 mm tungsten carbide beads (BioSpec Products, Inc.) in 100 μl PBS. ATP levels in larval homogenates were analyzed by CellTiter-Glo Luminescent Cell Viability Assay (Promega, Madison, WI) as reported previously [61].

### Pigment isolation

Pigment was extracted as described previously [62], with the exclusion of the urea treatment step. Briefly, worms were homogenised for 2 minutes using 1.1 mm beads, ultrasonicated for 5 minutes and spun down to collect supernatant. This was spun at 12,000g for 10 minutes and the supernatant aspirated out. The pellet was washed in 2.5% SDS and 0.1 M NaCO_3_ and incubated with proteinase K at 20 mg/mL at 37 °C overnight. The next day, the pellet was washed 3 times in SDS and 3–6 times in MilliQ water. Finally, the sample was lyophilised overnight.

### Pigment characterisation

Spectrophotometric absorbance of the pigment at 400 nm was evaluated on a pool of 100 iL3 dissolved for 24 hours in 0.1 M NaOH. All measurements were carried out in duplicate or in triplicate. Absorbance was read on a Tecan Infinite M1000 Pro plate reader.

Extracted peptides were subjected to mass spectrometry analysis as subsequently described. Eluted peptides were injected onto a 300 μm × 5 mm Agilent Zorbax SB-C18 trapping column and peptides were subsequently resolved on a 1.8 μm, 2.1 x 50 mm column containing Silica C18 packing.

### Homologue identification and phylogram analysis

BlastP (v2.2.28, https://www.ncbi.nlm.nih.gov/pubmed/18440982) was used to identify *Nb* protein sequences obtained from the L3 & “L5” adult secretomes [19] presenting high similarity to groups of selected target proteins of interest. BlastX (v2.2.28, https://www.ncbi.nlm.nih.gov/pubmed/18440982) was used to do the same for proteins translated from transcripts assembled from the iL3 transcriptome [19]. Top-scoring hits with alignments covering at least 95% of the *Nb* proteins were considered for further analysis. Two groups of target proteins of interest were defined: one including homologs of the *Na*-APR1 protein (see list below), and one including homologs of *Na*-GST1 protein (see list below). With these targets, 1 and 11 putative *Nb* protein homologs were manually identified from the Blast results, respectively. Proteins from each group were then aligned using Clustal Omega (v1.2.3, http://msb.embopress.org/content/7/1/539) with default settings for protein alignment. Multiple sequence alignments were visualised using the JalView 2 Desktop application (http://bioinformatics.oxfordjournals.org/content/25/9/1189). For the GST1 group, amino acids were visually coloured based on the ClustalX scheme. Molecular phylogenies were generated using Clustal Omega’s Phylogeny program using UPGMA clustering with distance correction (http://bioinformatics.oxfordjournals.org/content/23/21/2947.full), and were used to order sequences in the alignments. Finally, sequence features of interest were manually highlighted in the alignments.

### Statistical analysis

The choice of statistical tests was based on sample size and on Bartlett's test when normal distributions of the errors were expected. Data from separate experiments were pooled when possible. Total lung or gut worm numbers were analyzed by ANOVA (one- or two-way) or by t-test when only two groups were compared. Morphology parameters were analyzed using t-tests. Data were excluded only based on error of manipulation (incomplete worm injection). Representation and data analysis were performed with GraphPad Prism 5. Statistically significant values are indicated as follows: NS, P>0.05; * = P<0.05; ** = P<0.01; *** = P<0.001; **** = P<0.0001

### Ethics statement

All experimental procedures described in this study were approved under Protocol 2015R15 by the Victoria University Animal Ethics Committee in accordance with the the Code of Ethical Conduct for the use of Live Animals for Teaching and Research, Animal Welfare Act 1999 approved by the Ministry of Primary Industries, New Zealand.

## Supporting information

S1 Fig Protein-based neighbour-joining phylogram of *Nb*-GST-1 homologues. Amino acid alignment of *Nb*-GST-1 and the corresponding regions of other parasite and gluthatione transferase homologues. GenBank accession numbers are as follows: *N*. *americanus*, ACX53261.1; *A*. *duodenale*, L0EMQ0_9BILA; *Haemonchus contortus*, AAF81283.1. The sequence of *N*. *brasiliensis* putative GST-1 homologues (m.111632, m.154242, m.83139, m.28108, m.42690, m.161994, m.272028, m.428360, m.81737, m.135356, m.363040) were identified from the L3 transcriptome and are available elsewhere [19]. A protein-based neighbour-joining phylogram of several homologues from related organisms confirms the proximity of the *Nb*-GST-1 homologue to those of the hookworm family.
(TIF)

S2 Fig *Nb* hemozoin-like pigment is specific to haemoglobin consumption. (A) DIC image of *Nb* larvae harvested from the lung one day after infection, showing the characteristic intestinal pigmentation that appears in the molt 3 larvae. Data representative of three experiments, at least 50 larvae observed for each experiment. Scale bar: 50 μm. (B) 1500 iL3 are cultured *in vitro* for up to 4 days in the presence of 10^8^ mouse RBCs alone, or with varying doses of human transferrin, ferric citrate, human hemoglobin (Hb) or hemocyanin (Hc). The percentage of larvae harbouring intestinal pigmentation was counted daily for 4 days of stimulation. Data representative of three independent experiments, two-way ANOVA significant for both time and dose effect, Bonferonni post-test significant for all time points (but 0) relative to 10^8^ RBC.
(TIF)

S3 Fig Quinoline targeting of hemozoin in *Nb*. A) iL3 were either left unfed or fed *in vitro* with RBCs for 4 days (fed), or fed for one day followed by withdrawal (arrow) of the RBCs (fasted). The percentage of larvae with internal pigmentation was evaluated daily for 4 days. Data representative of three independent experiments (with triplicates of 1500 iL3 for each experiment), one-way ANOVA. (B) ATP measurement of iL3 treated *in vitro* for 4 days with or without CLQ and without RBC. iL3 boiled were used as a negative control, one-way ANOVA, Bonferonni post-test compared to untreated. (C) DIC image of an adult male harvested 6 days post-infection. The arrow indicates the intestinal pigment of the worm. Scale bar: 50 μm. (D) Absorbance at 400 nm of male or female adult *Nb* harvested from mice treated intraperitoneally for 6 days with QND (25 mg/kg) or vehicle alone. Data representative of two independent experiments (n = 5), one-way ANOVA.
(TIF)

S1 Video Red blood cell bolus movement in *Nb* intestine. RBC were isolated and stained with PKH26. Cells were then co-cultured at 1x10^8^ cells per 1500 iL3 for 24 hours after which larvae were assessed for internal fluorescence by wide-field imaging. Data representative of two independent experiments, with at least 50 larvae observed for each experiment.
(MOV)

S1 Text Sequence of *Nb*-APR-1 homologue. (DOCX)
