# Supplementary figures and images for: A novel blood-feeding detoxification pathway in Nippostrongylus brasiliensis L3 reveals a potential checkpoint for arresting hookworm development
Source: PLoS Pathog. 2018 Mar 22;14(3):e1006931. doi: 10.1371/journal.ppat.1006931 (PMC5864084; doi:10.1371/journal.ppat.1006931)

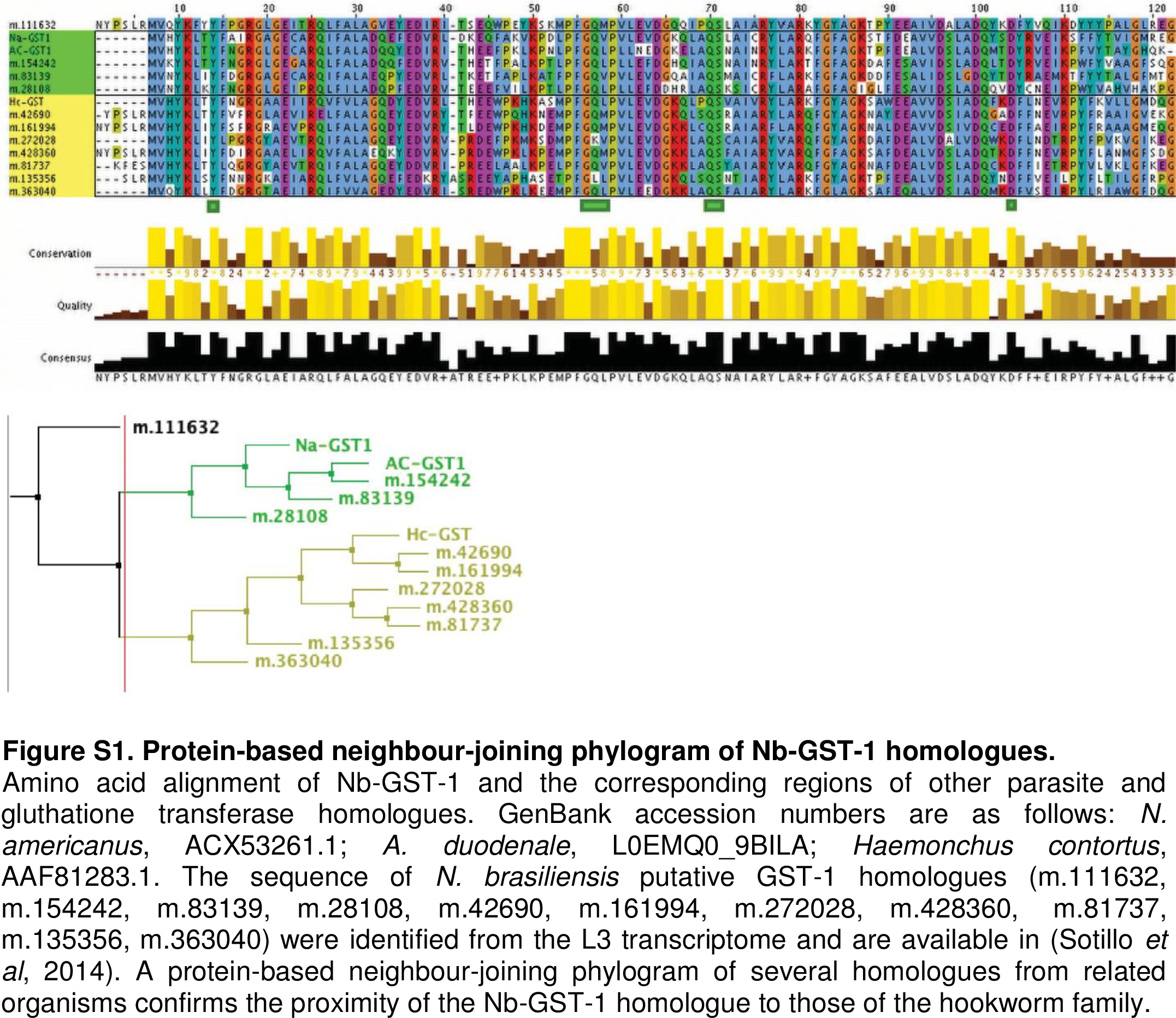

Supplement: S1 Fig — Amino acid alignment of Nb-GST-1 and the corresponding regions of other parasite and gluthatione transferase homologues. GenBank accession numbers are as follows: N. americanus, ACX53261.1; A. duodenale, L0EMQ0_9BILA; Haemonchus contortus, AAF81283.1. The sequence of N. brasiliensis putative GST-1 homologues (m.111632, m.154242, m.83139, m.28108, m.42690, m.161994, m.272028, m.428360, m.81737, m.135356, m.363040) were identified from the L3 transcriptome and are available elsewhere [19]. A protein-based neighbour-joining phylogram of several homologues from related organisms confirms the proximity of the Nb-GST-1 homologue to those of the hookworm family. (TIF) [file ppat.1006931.s001.tif]

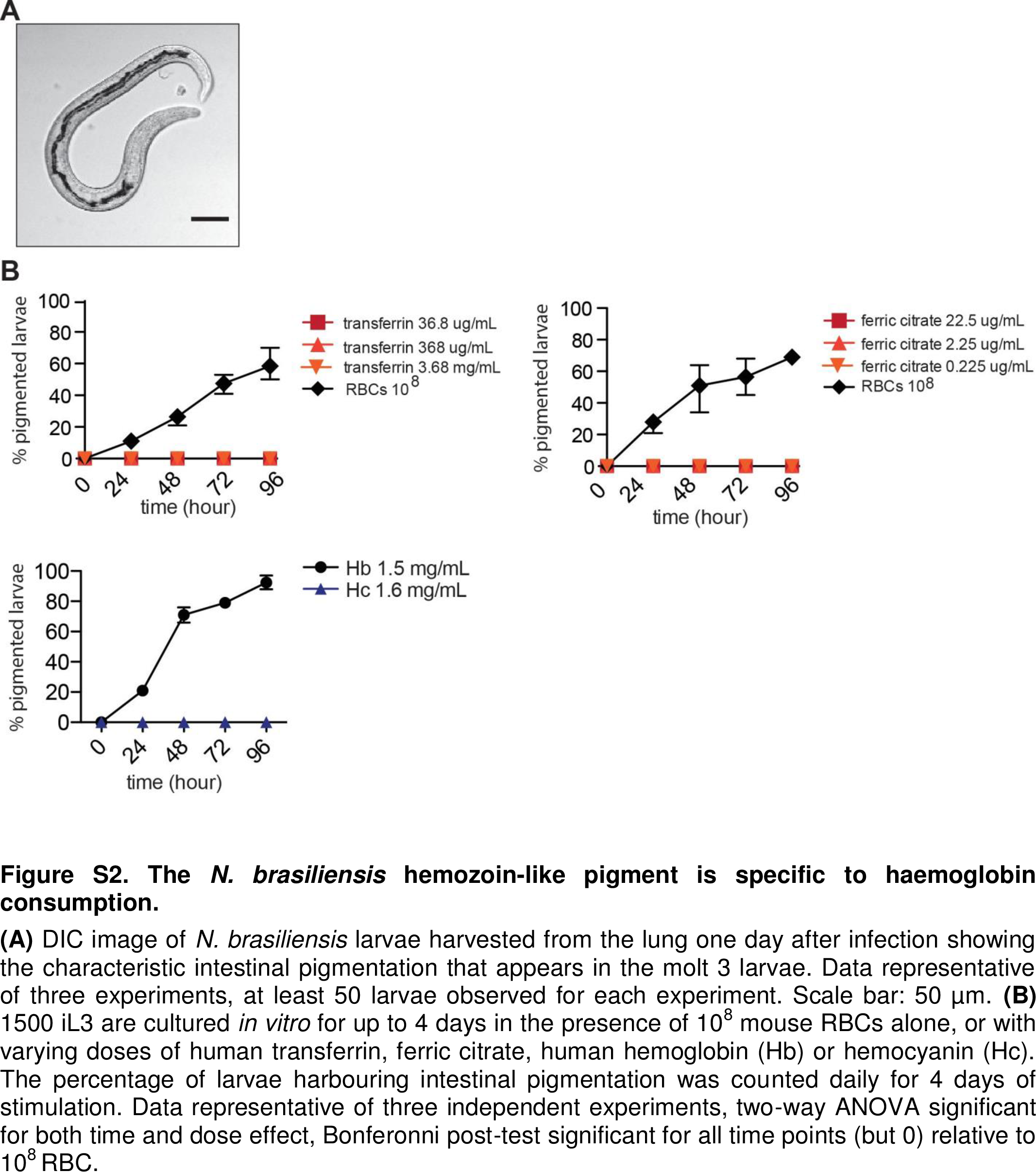

Supplement: S2 Fig — (A) DIC image of Nb larvae harvested from the lung one day after infection, showing the characteristic intestinal pigmentation that appears in the molt 3 larvae. Data representative of three experiments, at least 50 larvae observed for each experiment. Scale bar: 50 μm. (B) 1500 iL3 are cultured in vitro for up to 4 days in the presence of 108 mouse RBCs alone, or with varying doses of human transferrin, ferric citrate, human hemoglobin (Hb) or hemocyanin (Hc). The percentage of larvae harbouring intestinal pigmentation was counted daily for 4 days of stimulation. Data representative of three independent experiments, two-way ANOVA significant for both time and dose effect, Bonferonni post-test significant for all time points (but 0) relative to 108 RBC. (TIF) [file ppat.1006931.s002.tif]

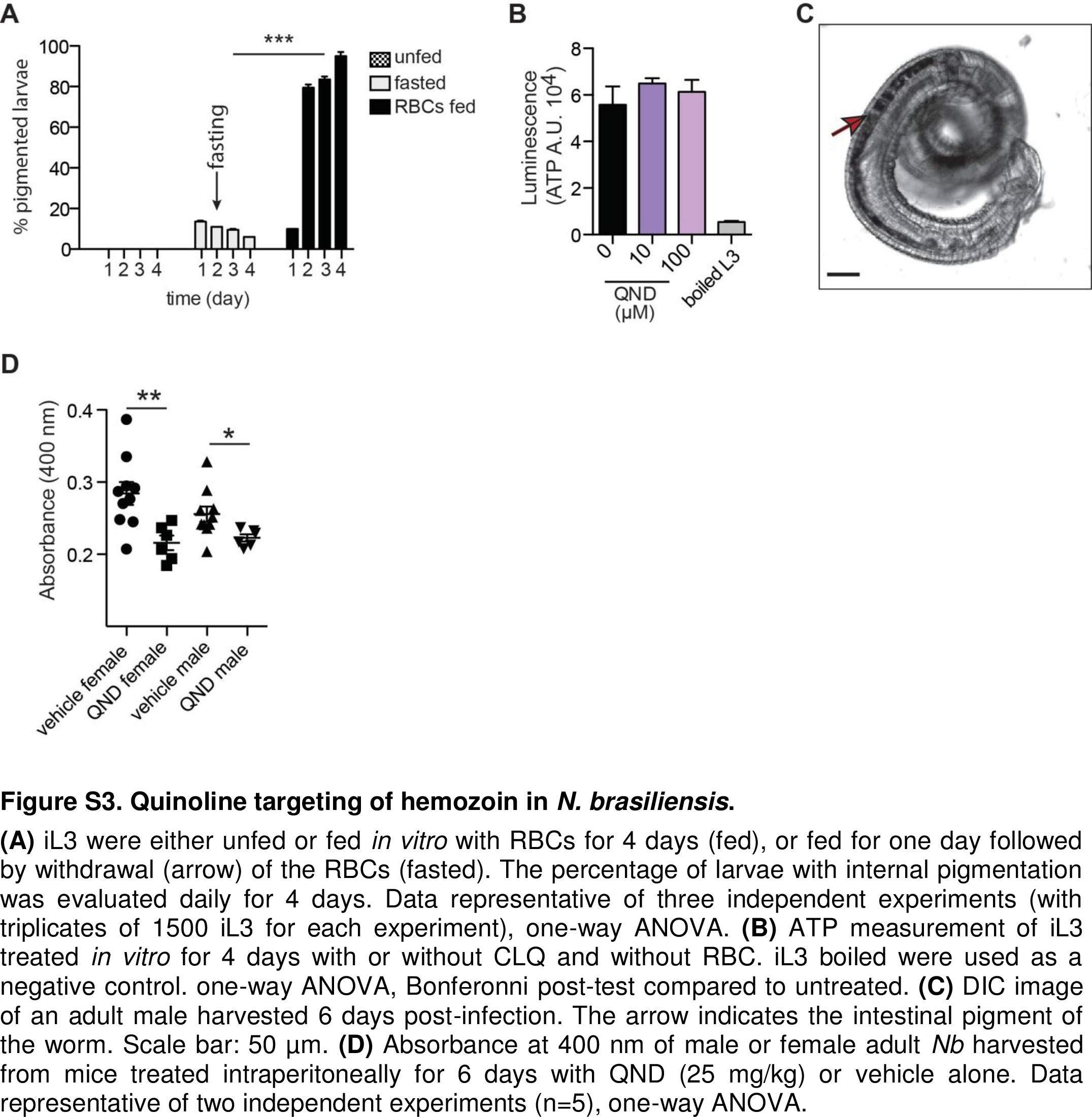

Supplement: S3 Fig — A) iL3 were either left unfed or fed in vitro with RBCs for 4 days (fed), or fed for one day followed by withdrawal (arrow) of the RBCs (fasted). The percentage of larvae with internal pigmentation was evaluated daily for 4 days. Data representative of three independent experiments (with triplicates of 1500 iL3 for each experiment), one-way ANOVA. (B) ATP measurement of iL3 treated in vitro for 4 days with or without CLQ and without RBC. iL3 boiled were used as a negative control, one-way ANOVA, Bonferonni post-test compared to untreated. (C) DIC image of an adult male harvested 6 days post-infection. The arrow indicates the intestinal pigment of the worm. Scale bar: 50 μm. (D) Absorbance at 400 nm of male or female adult Nb harvested from mice treated intraperitoneally for 6 days with QND (25 mg/kg) or vehicle alone. Data representative of two independent experiments (n = 5), one-way ANOVA. (TIF) [file ppat.1006931.s003.tif]
