# Supplementary material for: A novel blood-feeding detoxification pathway in Nippostrongylus brasiliensis L3 reveals a potential checkpoint for arresting hookworm development
Source: PLoS Pathog. 2018 Mar 22;14(3):e1006931. doi: 10.1371/journal.ppat.1006931 (PMC5864084; doi:10.1371/journal.ppat.1006931)
Supplement: S1 Text — (DOCX) [file ppat.1006931.s005.docx]

# Text S1. Sequence of Nb-APR-1 homologue.

>m.418883 AGLEGGQQTQEHYSHTARAPGGDMPRLLLLLALFAVGYGAIHHRRAYHSRRDVQSIPLTRQRTLRERLLLSGSWEDYQKQRNHYQKKLLAKYAANKAGKLQSTNEIDELLRNYMDAQYFGTIQIGTPAQNFTVIFDTGSSNLWVPSRKCPFYDIACMLHHRYDSGASSTYKEDGRKMAIQYGTGSMKGFISKDTVCVAGICAESQPFAEATSEPGLTFIAAKFDGILGMAFPEIAVLGVQPVFHTFIEQKKVPSPVFAFWLNRNPDSDLGGEITLGGMDARRYVDPITWTPVTRRGYWQFKMDTVKGGSSVVACPNGCQAIADTGTSLIAGPKAQVEAIQKYIGAEPLMKGEYMIPCDKVPSLPELTFVIEGKAFTLKGEDYVLSVKTGGKTICLSGFMGMDFPERIGELWILGDVFIGRYYTVFDIGEARVGFAQ AKAENGIPVPPAVREVRQYELFNTNSAEEDTFMNV
